# Forecasting future needs and optimal allocation of medical residency positions: the Emilia-Romagna Region case study

**DOI:** 10.1186/1478-4491-13-7

**Published:** 2015-01-30

**Authors:** Francesca Senese, Paolo Tubertini, Angelina Mazzocchetti, Andrea Lodi, Corrado Ruozi, Roberto Grilli

**Affiliations:** Regional Agency for Health and Social Care of Emilia-Romagna, Via Aldo Moro 21, 40127 Bologna, Italy; Department of Electrical, Electronic, and Information Engineering, University of Bologna, Bologna, Italy; Regional Bureau of Statistics of Emilia-Romagna, Bologna, Italy

**Keywords:** Health workforce forecast, Requirement model, Medical training, Optimization, System dynamics

## Abstract

**Objectives:**

Italian regional health authorities annually negotiate the number of residency grants to be financed by the National government and the number and mix of supplementary grants to be funded by the regional budget. This study provides regional decision-makers with a requirement model to forecast the future demand of specialists at the regional level.

**Methods:**

We have developed a system dynamics (SD) model that projects the evolution of the supply of medical specialists and three demand scenarios across the planning horizon (2030). Demand scenarios account for different drivers: demography, service utilization rates (ambulatory care and hospital discharges) and hospital beds. Based on the SD outputs (occupational and training gaps), a mixed integer programming (MIP) model computes potentially effective assignments of medical specialization grants for each year of the projection.

**Results:**

To simulate the allocation of grants, we have compared how regional and national grants can be managed in order to reduce future gaps with respect to current training patterns. The allocation of 25 supplementary grants per year does not appear as effective in reducing expected occupational gaps as the re-modulation of all regional training vacancies.

**Electronic supplementary material:**

The online version of this article (doi:10.1186/1478-4491-13-7) contains supplementary material, which is available to authorized users.

## Background

The medical labour market is predominantly public in Italy, as is university and residency training (*numerus clausus* was introduced in 1986). This supply constraint requires public regulation and accurate long-term planning [1]. Perceived shortages of medical specialists led to an increase of medical school intake in 2010 (+29%), although neither the current appropriateness of medical supply nor its likely evolution with regard to healthcare needs has been assessed. Conversely, in 2012, the central government downsized the number of residency training grants by 10% (from 5,000 to 4,500), passing onto the Regions further planning and funding responsibility for medical postgraduate training. The Regions correct perceived imbalances through the funding of supplementary grants that are negotiated annually with a number of interested parties (university and medical associations).

As the process outlined above is to a large extent implicit, the goal of this study was to develop a tool to support regional decision-makers in forecasting future demand for specialists. Accordingly, we have developed a simulation-optimization approach. A system dynamics (SD) model projects the supply of medical specialists and three demand scenarios across the planning horizon (2011–2030), while a mixed integer programming (MIP) model defines optimal allocation policies for residency vacancies.

Planning human resources for health (HRH) requires modelling long-term interplay between the supply of specialists and forecasting the changing needs of the population and the emerging care pathways. The need for long-term workforce planning and the availability of different approaches (supply, requirements, needs-based) have been stressed by the World Health Organization [2, 3], by the OECD [4] and recently by the launch of the EU Joint Action on Health Workforce Planning and Forecasting [5, 6]. HRH availability is crucial to pursuing high performance [7, 8] as HRH imbalances are associated with financial consequences [9], inappropriate demand for services, poor responsiveness to patient expectations due to burnout and waiting times, low quality and safety in cases of understaffing [10–13]. Defining appropriate staffing levels requires an agreement about ‘the right number’ [14] of professionals able to produce certain outputs, both at baseline and at any given point of the forecast; such a measure differs across health systems and by type of medical specialization; however, its extremes (scarcity or excess) are known to threaten health outcomes. Models for doctors’ supply and requirement forecasting have been developed in several countries: the USA [15], the Netherlands [16], Spain [17], Belgium [18] and Australia [19, 20]. No best practice exists as models are context-dependent, although they share common methodological issues. Researchers have debated the nuances and limitations of each model’s assumptions [21, 22] extensively, unfortunately sharing little practical experience on both technical and cultural barriers in the implementation of a quantitative method to forecast future HRH.

Available reviews on forecasting methodologies [23] typically lead the researcher through the dimensions characterizing the supply (age, gender, full-time equivalent, mobility and attrition) to pass onto the debate of how to *proxy* future requirements. This can be achieved by basic assumptions of compensating expected turnover due to retirement (*status quo* vs. changes in HRH productivity) or by building sophisticated assumptions on the dimensions affecting the demand for medical workforce: its possible drivers. The inclusion of epidemiological, social and normative information, the prerequisite of a needs-based approach [24, 25], and of economic variables into the forecasting model is strongly recommended, yet still infrequent in practice.

### Methodology

Our working hypothesis relies on the assumption that future Emilia-Romagna Region (ERR) HRH requirements and prioritization of regional grants have to be defined in correlation with foreseeable shortages, demographic and service utilization changes.

As we are dealing with a complex dynamic system, as the medical labour market is, we have opted for operational research techniques implementing a SD computer simulation model in Powersim Studio 9 (the model is shown in Additional file 1). The SD supply side model allows for the characterization, for each year of the projection, of the simultaneous behaviour of several stocks [26]. In our supply model, stocks represent aggregations of medical doctors, such as in-training and newly trained specialists, currently in the labour force (public, private, self-employed, general practitioners, district paediatricians), dealing with 43 specializations separately.^a^ On the demand side, the SD model enables the storage and display of data corresponding to each selected driver during the projection horizon (2011–2030). The assumptions and data sources of the model are summarized in Table 1 and are extensively described in the full report of this study [27].

**Table 1:** **Main supply and demand variables, assumptions and data source**

Variables			Assumptions	Data source
Supply side				
Inflow	Education	• No. of State-funded quotas 2012–2013	Constant at the 2012–2013 level (‘as is’ scenario)	MIUR
Stocks	Regional public NHS	• Headcounts of physicians by sex, age and specialization declared	Appropriate at 2011 levels for all stocks	Regional databases
	Private (no. 1,021)			*Ad hoc* survey
	Conventioned (GPs, district paediatrics)		According to recommended population ratios	Normative
Outflows	Regional public NHS	• Sex-, age- and specialization-specific exit rates	Leaving the regional NHS due to retirement, shift to the private sector and move to other regions before retirement. Observed exit rates in 2001–2011 by cause apply each year	Regional databases of 2001–2011 observations
	Private sector and self-employed personnel	• Age-specific exit rates	Females leave at 67 and males at 70	Normative
Demand side				
Population	Demographic projections	• Sex, 5-year band population projections to 2030	Central scenario	Regional statistics bureau
Service utilization	Outpatient activities (ASA) plus hospital discharges (SDO) by specialization provided to patients between 2002 and 2011	• Patients’ sex, 5-year bands, consumption rates by specialization	2002–2011 outpatient and inpatient utilization rate trend line extrapolation and projection to 2021. Expected regional age/sex cohorts will consume more or less of each specialization service and a different mix of outpatient visits and hospital discharges	Regional databases (ASA, SDO)
Hospital beds	Public hospital beds by specialization at 2011	• No. of public hospital beds and optimal staffing standards per specialization	Physician-to-hospital bed standards define the future requirement of specialists	National guidelines

### Supply side assumptions and data

The supply representation of a medical specialist in Italy begins with admission to specialization schools, whose courses last between 4 and 6 years. Once training is completed, newly trained specialists become ‘available’ in our model and can access the public or the private sector (inflows). As we aim to model future training requirements for the whole regional medical labour market, we widened the spectrum of possible employment sectors to include:

-  Public sector (Regional NHS)
-  Private hospital sector (AIOP)
-  Self-employed HRH contracted by the Regional NHS: ambulatory specialists, general practitioners, district paediatricians.

Data collection and data mining operations were complicated by the multiple sources and their lack of homogeneity. Regional administrative databases were designed to answer specific organizational requirements (mainly transparency) but fail to support HRH planning. Our supply representation of medical stocks in 2011 followed the classification provided by the Ministry of Education (MIUR) [28]. This information was available about public-employed specialists, and it was also acquired for doctors working in 23 private clinics (out of 48 active in ERR). Inflow rules were quite basic: the number of national residency positions assigned to ERR will replicate academic year (a.y.) 2012–2013 patterns and will produce 451 trainees a year, aged between 31 and 36 with a gender divide assumed to be as observed in 2011. At baseline (2011), the model includes enrolled trainees who began in a.y. 2007–2008, becoming available at year 1 of the projection. This is our ‘*as is*’ scenario with regard to future specialist training at the regional level.

Since regional databases do not register any employee’s motivation for leaving work, outflow rules for the public sector were defined by observing 10 years of doctors’ turnover and appearance across different databases. By observing 2001–2011 in/outflows from public registers, we built up failure rates attributable to i) retirement, ii) pre-retirement age passage towards the private sector (this occurs when a doctor opens a VAT position, incompatible with NHS employment) and iii) pre-retirement exit from the regional labour market due to other reasons (doctor NHS identification code or VAT position lost due to leaving or migration). As these are mutually exclusive events, a competing risks model [29] was used to estimate specialization-age-sex-specific failure rates for the three exit causes. This was done to account for the high dynamism observed, especially among male surgeons in the 45–65 age band in the public sector towards the private sector, which is known to drain skilled physicians from the public sector. Specialization-specific attrition rates also account for the relatively higher pace of turnover among certain specializations (e.g. Anaesthesiology) with respect to more stable ones (e.g. Geriatrics, Psychiatry). The drain of public HRH towards the private and self-employment sectors should gradually ‘saturate’ these markets and mitigate their demand scenarios to 2030. Exit rules from private and self-employed medical stocks were, for lack of reliable retrospective data, kept relatively simple and assumed that males leave the profession at 70 and females at 67 (normative figures).

Finally, we implemented the supply model for specialists active in 2011 in ERR in the SD software. The training and working life cycle deals with physicians of 43 different profiles, aged between 26 and 70, divided by gender and belonging to one of the interlinked employment sectors.

### Demand side drivers and assumptions

The projection of future workforce demand is a task of great complexity due to the arbitrary definition of drivers of the current and future demand. However, demand is often defined taking into account population, expected service utilization changes due to organizational and technological improvements and labour market dynamics. Each of the above dimensions is bound to a combination of drivers and expressed through a specific physician-to-driver ratio or benchmark; in particular, the following three drivers were chosen:

*Population demographic projections to 2030*: Demographic change is undoubtedly relevant in predicting future service utilization, although it is not the only one. Migration flows, fertility rates and ageing will affect future service demand and, consequently, staffing requirements. In the demand model, we included the regional population projections, sex-age specific to 2030, developed by the regional statistics bureau in 2011 [30]. Physician-to-target-population ratios are assumed to be appropriate at baseline (2011) and do not vary over the projection horizon. Some specializations, such as Gynaecology and Obstetrics, Geriatrics, Infant Neuropsychiatry and Paediatrics, are clearly bound to specific population segments.

*Ambulatory visits and hospital discharges by discipline* (service utilization driver): Given the lack of well-defined physician-to-epidemiological-condition ratios, healthcare pathways’ staffing requirements can be defined as the ratio between the volume of activities and the type of specialist involved. We retrieved information on hospital discharges and outpatient services (diagnostic appointments, treatment and rehabilitation) provided to the population between 2002 and 2011 and used them to proxy a physician requirement model [31]. Service utilization data for public and private inpatient and outpatient appointments are recorded by two regional databases: hospital discharge records (SDO) and ambulatory specialist consultations (ASA). Most ASA and SDO records can be attributed to a specific discipline, providing a valuable indicator of specialization-specific resource utilization by the population. As a starting point, we analysed how service utilization by the resident population (5-year age/sex-specific bands) has changed for inpatient and outpatient activities in the last decade. In the chosen period, a general reduction of public hospitalization occurred; a drop of inpatient services was recorded for males and females in the 70–74 age band (-22.5% and -21.9%, respectively), while a reverse trend occurred in the 0–5 age band (+69.4% and +111.1%, respectively) for surgical specialties. A decrease in inpatient activities was compensated by an increase in outpatient activities, particularly significant for the Medical area. By observing a decade of in/outpatient service utilization, we extrapolated specific trend lines for each ‘discipline-sex-age’ combination up to 2030. These trend lines are projected until 2021 and then a binding factor is considered until 2030. In this way, the model ought to account for increasing de-hospitalization of some procedures and for latent shifts in productivity, due to technological changes, which we were not able to define systematically for all 43 profiles. In the self-employment and private sectors, doctors are likely to attend a clinic only a few hours a week and to carry out consultations in their surgeries for which no registry exists. Therefore, medical HRH working outside the public system are linked only to population projections in our model. Combining the population projections and the service utilization trend lines for 2011–2030, we obtain the expected volume of in/outpatient activities, which hence return the expected number of specialists required to provide them.

*Hospital beds*: The number of beds assigned to a given specialization is a ‘chokepoint’, a structural constraint that is commonly used to estimate the optimal staffing levels. This indicator can be considered appropriate when most of the activities involving a specific specialization are related to hospitalization; this is particularly true only for Medical specializations where peri-operative activities are a key aspect and for some Surgical specializations. However, we included this driver because the public spending review of the Italian NHS explicitly stresses the reduction and conversion of public beds. Hospital beds assigned to each discipline can therefore be considered as a constraint to which the future number of public medical specialists can be bound. The bed-driven scenario exploits the staffing standards developed by national experts to be applied to Regions that run a healthcare deficit [32]. In these guidelines, physician-to-bed ratios vary between 0.24 head per bed for low-complexity specializations to 1 for intensive care.

The selected demand drivers can be combined in the SD projection and provide three HRH requirement scenarios:

-  Scenario 1: population-driven demand
-  Scenario 2: inpatient- and outpatient-driven demand
-  Scenario 3: hospital core (bed standards) and increasing outpatient consultation demand.

## Results

### Occupational and training gaps: three demand scenarios vs. ‘*as is*’ national residency training

**Figure 1:**
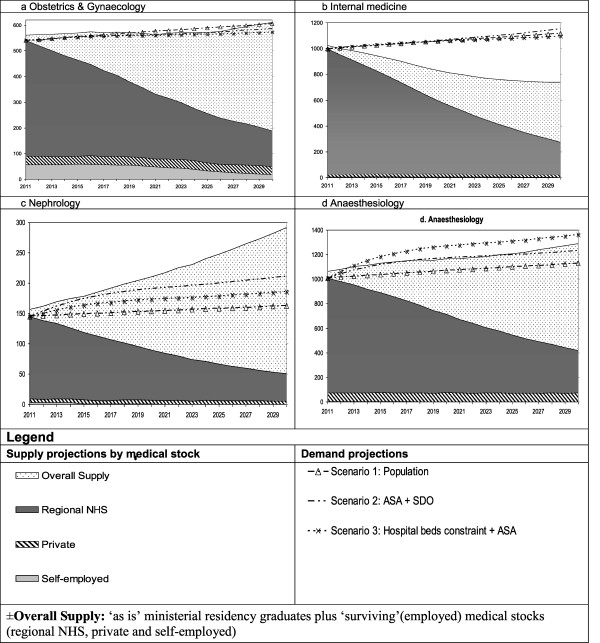
**Supply and demand projections to 2030 for selected specialties. (a)** Obstetrics and gynaecology. **(b)** Internal medicine. **(c)** Nephrology. **(d)** Anaesthesiology.

The SD graphic outputs are as shown in Figure 1a–d. The first scenario (triangle line) returns an average increase in demand of +12% by 2030 with respect to the medical stocks at baseline (Table 2). Scenario 2 (dotted line) leads to the highest increases in demand, especially for medical specializations and for those serving growing segments of the population (0–14 years old and over 65). The scenario that binds specialists to hospital beds (asterisk line) is either conservative or mildly increasing, as it accounts for a portion of professionals who will provide outpatient consultations as well as hospital bed visiting activities (SDO). The dotted area in the graph is fed by newly trained doctors according to the ‘as is’ scenario of national grants assigned to the ERR and represents the overall supply in a given year. For each year of the scenario, in fact, neo-graduates are added to professionals expected in the labour force, generating the overall regional supply, which creates a surplus or a deficit (gap) with respect to the three demand scenarios. The right-hand columns of Table 2 show whether the ‘*as is*’ scenario of national funded grants repeated between 2012 and 2024—last year in which a decision regarding a 6-year training will affect the 2030 training gap—satisfies the three demand scenarios. For instance, a perpetuation of ministerial grant assignations as of 2011 appears not to meet any of the expected requirements for Internal Medicine (Figure 1b), Ophthalmology, Neurosurgery, Psychiatry, Emergency and Internal Medicine (Table 2). Should scenario 2 (ASA + SDO) prove to be correct, a deficit in Anaesthesiology (Figure 1d), Otolaryngology, Urology and Cardiology would also occur. As expected, scenario 3 predicts a lower rate of increase of specialists involved in surgical procedures compared to scenario 2, with the exception of Neurosurgery, for which 2011 staffing levels were found to be too low when applying the physician-to-bed ratio equal to 1, as prescribed by the selected standards [32].

**Table 2:** **Demand increase at 2030 by scenario and training gaps w.r.t. national residency training policy**

Area	Selected specialization	Stock at 2011 ^a^	Demand % increase at 2030			Training gaps in 2030 w.r.t. ‘as is’ national grants		
			Scenario 1	Scenario 2	Scenario 3	Scenario 1	Scenario 2	Scenario 3
Surgical	General surgery	580	12.0	12.6	8.8	49	46	68
	Gynaecology and obstetrics (Figure 1a)	540	12.0	8.5	6.1	19	38	51
	Neurosurgery	93	12.0	18.7	53.1	-34	-40	-45
	Ophthalmology	322	12.0	18.3	17.4	-76	-96	-93
	Orthopaedic and trauma	623	12.0	15.4	7.8	81	60	107
	Otorinolaringoiatry	201	12.0	19.3	12.3	1	-14	0
	Urology	191	12.0	21.5	7.1	3	-15	13
	Cardiac surgery	44	12.0	13.8	6.9	53	53	56
Medical	Geriatrics	237	20.3	31.2	16.4	46	20	55
	Internal medicine (Figure 1b)	997	12.0	15.4	9.9	-344	-378	-323
	Emergency medicine	595	12.0	10.6	10.0	-260	-252	-248
	Infant neuropsychiatry	171	17.5	36.7	37.9	2	-31	-33
	Psychiatry	590	11.1	4.5	3.6	-63	-25	-19
	Gastroenterology	159	12.0	34.3	25.9	31	-5	9
	Cardiology	553	12.0	40.0	15.6	101	-54	81
	Respiratory diseases	163	12.0	27.4	17.9	85	60	75
	Nephrology (Figure 1c)	145	12.0	45.9	28.1	129	80	106
	Rheumatology	36	12.0	34.8	34.3	64	56	56
Services	Anatomopathology	130	12.0	15.4	0.0	58	54	74
	Radio-diagnostics	729	12.0	32.4	32.3	148	-1	1
	Radiotherapy	68	12.0	41.3	40.6	115	95	95
	Anaesthesiology (Figure 1d)	1,009	12.0	35.2	22.2	171	-63	69
	Physical and rehabilitation medicine	262	12.0	2.4	1.7	24	49	51

^a^Includes all the medical stocks: public, private and self-employed outpatient specialists.

### An optimal allocation model for regional funded residency grants

Once training gaps are measured, priority rules for the optimal allocation of regional supplementary residency grants should be agreed upon. The rationale for setting up an allocation tool is that long-term planning requires accounting for the structural impact of annual decisions taken over the planning horizon, instead of relying on perceived annual shortages. The proposed MIP model was set to minimize the overall regional training gaps until 2024 by considering the length of each residency programme and some weighted criteria. We implemented the optimization model with CPLEX Optimization Studio 12.5 (IBM).

The allocation of residency grants can be based on two different assumptions:

I. That ERR supplements nationally funded grants by funding 25 extra grants a year;
II. That regional managers can influence national allocation policies regarding regional training. The policy variable ‘total residency positions’ becomes a function of the estimated required HRH.
I) The regional health authority can fund and allocate a finite number of specialization grants (average 25 per year) among the regional medical schools. When deciding the grant mix to be sponsored, it is important to define the constraints and the prioritization criteria.

The allocation of funded grants is constrained by i) estimated requirements (training inflows cannot exceed time-lagged demand projections), ii) a budget which ultimately defines the maximum number of grants that can be allocated to the specialization schools each year and iii) a binding factor of maximum five grants per school to avoid excessive drainage by a specialization with higher training capacity and higher need.

Among the feasible solutions that satisfy the above constraints, the MIP model retains the ones that best respond to the three following prioritization criteria:

*P1*: Complexity of care index (intensive: 2.25, high: 2, medium-high: 1.75, mean: 1.50, medium-low: 1.25, low: 1, predominantly outpatient setting or services: 0.50);

*P2*: Absorption by the public sector (% of total specialists employed by the Regional NHS);

*P3*: Magnitude of the demand gap compared to the 2011 supply.

The first criterion evaluates the impact of complexity of care and solves several trade-offs among concurring specialization gaps in any given year, since the specializations defined as having intrinsic higher complexity of care gain allocation priority. The second prioritization factor considers the absorption of each specialization curriculum by the public sector. The occupational range within the public sector is meant to account for two phenomena: on the one hand, it should protect the interests of the public sponsor, thus improving its return on investment in supplementary training, while on the other hand, it should prevent the widening of gaps in those specializations mainly found in the public sector. No private market exists for some specializations that serve essential health needs: Paediatrics, Neonatology, Emergency Medicine and Psychiatry. The third factor (P3) normalizes the forecasted gaps so that specializations that have fewer staff do not get overlooked in favour of the more numerous ones. The parameter settings for both constraint (iii) and the complexity of care weights (P1) are discretionary choices made after running model sensitivity tests and in-depth discussion with parties involved. For instance, as for constraint (iii), one request was to maintain a reasonably realistic training capacity without excessive allocation of grants in a given year. Furthermore, when different constraints were tested, fewer grants per school led to less effective allocation (more schools received grants, yet gap mitigation was slower), while allowing more grants per school was more effective in the short term but led to implausible intermittent training activities. Prioritization according to the complexity of care responded to a classification provided in [32], which was translated into a scoring system whose weighting aimed to offset the risk of excessive requirements by low complexity and what we call ‘predominantly outpatient setting or service’ specializations, such as Laboratory Medicine, Preventive Medicine and Pathology.

The mixed integer linear programming model, which is presented in detail in [33], returns year-specific and cumulative allocations as shown in Table 3.

**Table 3:** **Cumulative allocations by main medical class (surgical, medical, services)**

Area	Number of grants (2012–2024)		
	Scenario 1	Scenario 2	Scenario 3
Surgical	73	71	98
Medical	239	277	237
Services	36	0	13
av.	348	348	348

Table 3 summarizes the cumulative allocation of 25 supplementary grants per year (23 in 2011 and 25 from 2012) for the 2011–2024 period, aggregating schools according to their disciplinary area. It is clear that, irrespectively of the demand scenario, additional grants would be allocated to Medical area specializations, while the Service area receives a smaller number of supplementary grants due to national input being adequate for this area, along with a lower index of complexity of care associated with these specializations. Cumulative allocations by specialization [33] suggest that Emergency Medicine is the priority, requiring between 22% and 34% of supplementary grants depending on the demand scenario. Other priority specializations are Paediatrics, Internal Medicine (scenario 1), Psychiatry and Neuropsychiatry. The last two receive a large number of grants due to different factors, namely psychiatrists will be strongly reduced due to retirement rates (stock at baseline is quite old), while Neuropsychiatry will be affected by a significant expected increase in the demand of ambulatory consultations for a (mildly increasing) infant population. In conclusion, it is important to note that the allocation of 25 additional grants does not appear to be sufficient to close the overall regional training gaps.

II) Grant funding can also be discussed as the hypothetical allocation of the overall regional residency grants according to future requirements in contrast to the ‘*as is*’ national funded grant scenario. In this case, the allocation model is characterized by the pooling of the regional and national budgets devoted to this task, which generates a theoretical allocation of 518 grants in 2011 plus 476 till 2024 (cumulative 6,706 grants). The grant requirements are instead unconstrained, which means that each specialization grows according to the three demand scenarios. The national allocation of grants is influenced by the number and the size of regional university hospitals and existent training capacity, which have not undergone major changes in the last few decades. It is therefore important to note that the theoretical allocation of 6,706 cumulative grants with the primary objective of reducing occupational gaps could lead to the drastic compression, or temporary suspension, of some medical schools during the forecast horizon.

Even though it is beyond the scope of this study to recommend a radical reorganization of regional training centres, it is interesting to analyse how greater coordination of national and regional funded grants could eventually satisfy the demand scenarios while leading to a budget curb.

As we can observe in Table 4, scenario 2 absorbs the maximum number of grants (6,706) and would require additional 2.9% grants funding in order to satisfy the estimated grant requirement (6,900). An allocation that satisfied the first scenario requirement (population) would lead to a modest integration (3%) of the ‘*as is*’ national funded pattern and would not fully exploit the supplementary grants as, already in 2024, the training budget could be reduced by 2.4%. The allocation of 6,706 cumulative grants would also be sufficient to satisfy the third scenario, leading to savings of 1.1% by 2024.

**Table 4:** **The results of the cumulative allocation of all grants according to the three demand scenarios vs. ‘as is’ allocation**

Area	Grant requirement (2012–2024)			‘as is’ scenario	∆% w.r.t. ‘as-is’ scenario		
	Scenario 1	Scenario 2	Scenario 3		Scenario 1	Scenario 2	Scenario 2
Surgical	1,425	1,519	1,364	1,617	-11.9	-6.1	-15.6
Medical	3,340	3,814	3,398	2,743	21.8	39.0	23.9
Services	1,782	1,567	1,871	1,998	-10.8	-21.6	-6.4
Tot.	6,547	6,900	6,633	6,358	3.0	8.5	4.3
∆% w.r.t. 6,706^a^ allocations	-2.4	2.9	-1.1	^_^	^_^	^_^	^_^

^a^Cumulative funded grants at 2024: 518 (495 + 23) funded in 2011 plus 476 (451 + 25) from 2012 to 2024.

Table 4 also shows the significant shift of grants from Surgery and Service area towards Medical specializations (min 21.8%—max 39%), as the result of the combination of wider demand gaps, higher care complexity and expected increase of inpatient and outpatient service utilization by the population.

A deeper analysis of the national and regional grant allocation by specialization, provided in [27] in Italian and in [33] in English, underpins the scarcity, in absolute terms, of the current grants assigned to Emergency and Internal Medicine. Conversely, the future needs of Plastic Surgery, Sports Medicine, Vascular and Thoracic Surgery as well as Respiratory System Diseases seem overestimated by current training policies. It is worth underlining that for ‘grant requirements’ in Table 4, we also include those specializations for which no training is available at the regional level, meaning that, by allocating grants according to estimated needs, some budget would then be available to support residency training outside Emilia-Romagna. The most interesting finding is that, surprisingly, for two out of three scenarios, the current budget devoted to residency training seems adequate to satisfy future specialist requirements. This means that the foreseeable baby-boomers retirement, mainly observable in the regional NHS, can be managed without a dramatic impact on public funds dedicated to HRH training if national imbalances in grant allocation are prevented.

## Discussion

For the first time, the SD model provides a comprehensive overview of regional data availability and it reveals the level of accuracy that a regional quantitative approach to HRH can achieve. The main contribution of the proposed approach is the systematic presentation of Italian regional supply and demand variables to support training choices. The long-term perspective chosen (2030) not only covers the length of postgraduate medical training, but, thanks to the integration of SD with the MIP model, it also displays the impact of allocation policies—when future supply and demand scenarios diverge—and, not least, the course of inaction. Before this study was undertaken, there was a lack of information management and no systematic representation of the regional health workforce sector. The overall—yet inevitably incomplete—picture of the active labour force belonging to different stocks at baseline (2011) was crucial to avoid stumbling into the assumption of regional medical training self-sufficiency as a mere response to public sector turnover.

Training decisions are effective after 5 or 6 years, by which time different employment sectors will be competing to hire trained physicians. Up to now, training policies have been based on the results of annual surveys with public local health trusts and on occasional consultations with other stakeholders which appraised future imbalances based on current staffing levels and expected retirements, without accounting for mobility flows towards the private and self-employed sectors. The estimate of two different concurrent motives for leaving the Regional NHS before retirement age aimed to account for a certain amount of dynamism in the system, even though data inconsistency could lead to outflow misestimations. We have also tried to address future changes in service utilization by extrapolating trend lines from 10-year observations and by linking these forecasts to future population projections. Using services as a proxy of population health need has its shortfalls as longitudinal analysis suggests that different population cohorts not only develop different health problems and have different unmet needs, but they also demand different services. Nevertheless, by projecting population shifts in service utilization from inpatient to outpatient consultations, we hypothesize that further de-hospitalization pushes will occur, as recommended by national and regional guidelines.

Limitations in our data analysis were due to the lack of full-time equivalent information, which is unreliable or incomplete in the databases considered. In addition, no information is included on the proportion of workload required by outpatient activities compared to inpatient activities; both are count variables at present. Unemployment at baseline is unknown, and underemployment is modelled as a positive difference between supply and demand of trainees in a given year. Mobility of health professionals to and from other regions is also underestimated. In fact, our model simulates high attrition rates towards the private regional sector and towards other regions, but it does not account for inflows other than new regionally trained physicians. Another possible study limitation is the lack of inclusion of an economic constraint in the requirement scenarios for the public sector, such as total salaries payable by the public budget in the coming years. However, as for other modelling choices (central population growth scenario, higher attrition rate before retirement and unbound service utilization trend lines to 2030), we have allowed for physician demand to expand, rejecting any *status quo* supply assumption. The idea was to create conceptually plausible scenarios, but inflated, to account as much as possible for population needs.

Further modelling efforts should be devoted to assessing current imbalances (surplus or deficit) in regional HRH with respect to the services being offered at baseline. This can be achieved by monitoring some indicators of workforce imbalances [34]. However, we believe a major improvement could be achieved by involving medical specialists and relevant stakeholders more systematically in the forecasting exercise; as a matter of fact, our group of experts was consulted at different stages of the research but could have played a stronger role in the definition of the plausible scenarios. Expert opinion is crucial to agreeing upon staffing standards, within activities by specialization and current supply appropriateness.

## Conclusions

Emilia-Romagna will face important challenges in the management of its medical supply. Current training strategies, both national and regional, may be unsatisfactory in the face of high negative turnover phenomena. In addition, population demographic trends will place higher stress on specializations related to the elderly. This is widely acknowledged, yet we have shown that an ageing population will not affect all medical specialist demand equally. For some specializations, the three scenarios highlight a foreseeable lack of trainees that could be curbed simply by redefining both national and regional allocation choices. The model suggests that 25 supplementary regional grants will not be enough to cope with future system shortages, while the shortages estimated by scenarios 1 (population) and 3 (hospital bed constraints) could be compensated before 2024 should all available training vacancies be planned according to our projections.

Because of the aforementioned limitations and the inner complexity of the medical labour market, it is clear that no simulation-optimization outputs can be considered as exact forecasts, and this study is no exception. Our study suggests that the main barriers to quantitative HRH modelling are data mining efforts and the choice of appropriate demand drivers. We found no mixed integer programming approach in the literature to provide an optimal quantitative answer to the common problem of planning future residency training. The classification of specializations according to their expected demand increase, their classification according to the index of complexity of care and their public vs. private occupational range offers new grounds for discussion for our experts and regional representatives. Notwithstanding its intrinsic limitations, our study is the first quantitative and systematic attempt in the Italian context to define a comprehensive methodology for strategic planning and forecasting of HRH.

## Endnote

^a^There were 60 specializations recognized by the Ministry of Education (see [28]), although we only modelled those employed and declared at the time of the study. For instance, the following are not included: Dentistry, Oral Surgery, Aerospace Medicine, Criminal Psychology, Thermal Medicine, Audiology, Neurophysiopathology, Medical Statistics and some other less common ones.

## Electronic supplementary material

Additional file 1: **System dynamics simulation model.** The file illustrates the stock and flow model for medical doctors supply and demand in the Emilia-Romagna Region, showing stocks interactions. (PDF 19 MB)
