# Supplementary figures and images for: Forecasting future needs and optimal allocation of medical residency positions: the Emilia-Romagna Region case study
Source: Hum Resour Health. 2015 Jan 30;13(1):7. doi: 10.1186/1478-4491-13-7 (PMC4328064; doi:10.1186/1478-4491-13-7)

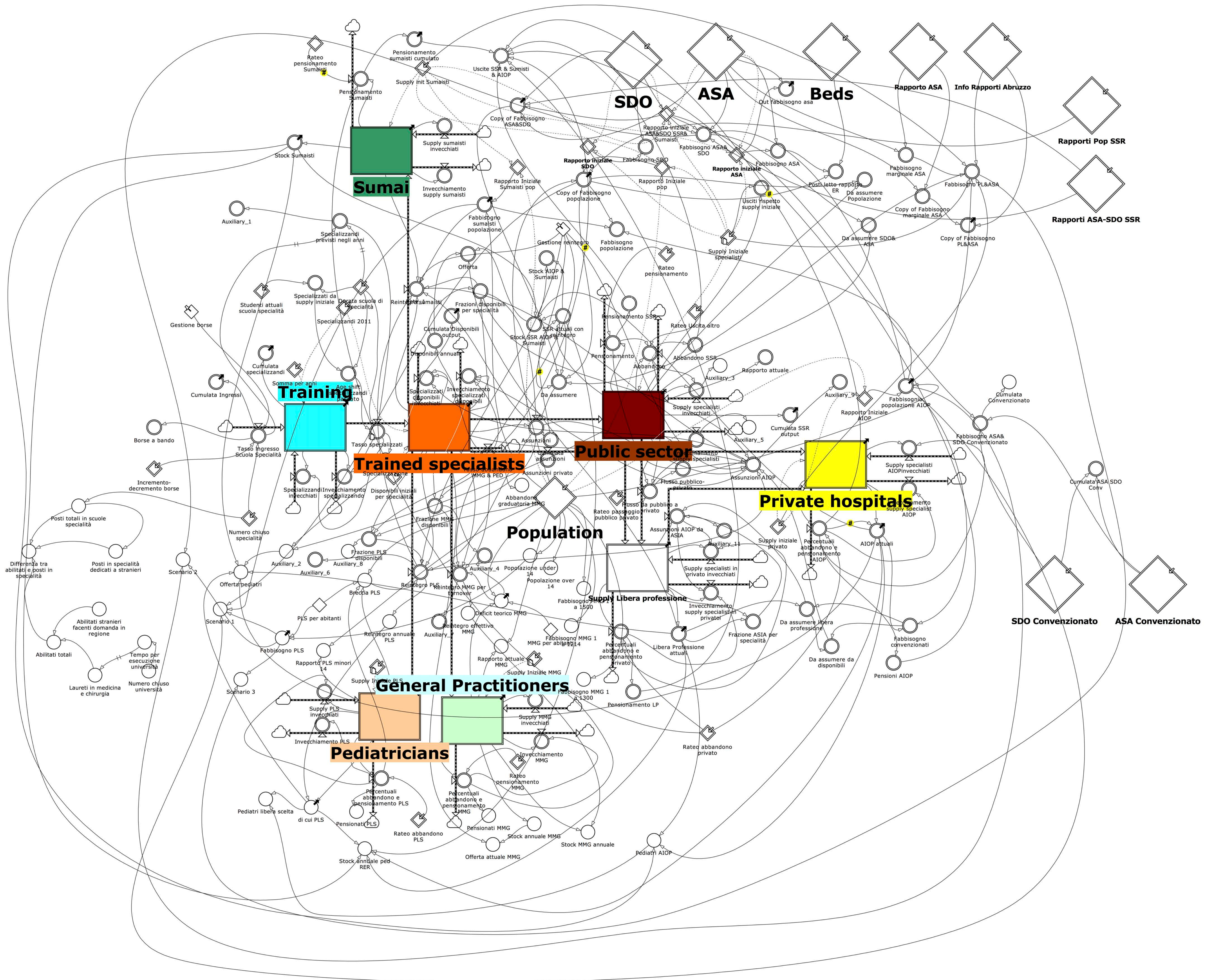

Supplement: Supplementary file 1 — Additional file 1: System dynamics simulation model. The file illustrates the stock and flow model for medical doctors supply and demand in the Emilia-Romagna Region, showing stocks interactions. (PDF 19 MB) [file 12960_2014_474_MOESM1_ESM.pdf]
